# Body Appreciation as a Factor Associated with College Students’ Willingness to Receive Future COVID-19 Vaccines

**DOI:** 10.3390/vaccines9111285

**Published:** 2021-11-05

**Authors:** Zi-Han Liu, Wei Bai, Hong Cai, Shou Liu, Xu Chen, Han Qi, Rui Liu, Teris Cheung, Zhaohui Su, Todd Jackson, Sha Sha, Yu-Tao Xiang

**Affiliations:** 1 Unit of Psychiatry, Department of Public Health and Medicinal Administration, & Institute of Translational Medicine, Faculty of Health Sciences, University of Macau, Macao 999078, China; yb97603@umac.mo (Z.-H.L.); yc07611@connect.um.edu.mo (W.B.); yc07640@umac.mo (H.C.); ytxiang@um.edu.mo (Y.-T.X.); 2 Centre for Cognitive and Brain Sciences, University of Macau, Macao 999078, China; 3 Institute of Advanced Studies in Humanities and Social Sciences, University of Macau, Macao SAR, Macao 999078, China; 4 Department of Public Health, Medical College, Qinghai University, Xining 810000, China; liushou@qhu.edu.cn; 5 The National Clinical Research Center for Mental Disorders & Beijing Key Laboratory of Mental Disorders Beijing Anding Hospital & the Advanced Innovation Center for Human Brain Protection, Capital Medical University, School of Mental Health, Beijing 100069, China; chenxuadyy@ccmu.edu.cn (X.C.); qihan@ccmu.edu.cn (H.Q.); ruiliu@ccmu.edu.cn (R.L.); 6 School of Nursing, Hong Kong Polytechnic University, Hong Kong 999077, China; teris.cheung@polyu.edu.hk; 7 Center on Smart and Connected Health Technologies, Mays Cancer Center, School of Nursing, UT Health San Antonio, San Antonio, TX 73301, USA; szh@utexas.edu; 8 Department of Psychology, University of Macau, Macau 999078, China; toddjackson@um.edu.mo

**Keywords:** body appreciation, COVID-19 vaccine, vaccination intentions, college student

## Abstract

Background: Following the initial manufacture of COVID-19 vaccines, numerous studies have investigated factors that influence people’s vaccination intentions. However, no studies have examined links of vaccination attitudes with body-related attitudes, especially body appreciation. To address this gap in the literature, we conducted this study to disentangle the relationship between college students’ COVID-19 vaccination intentions and body appreciation. Method: A cross-sectional study was conducted among Chinese college students. Participants completed the Body Appreciation Scale-2 (BAS-2) and other questionnaire measures of demographics, intentions to be vaccinated, and attitudes toward COVID-19 vaccination programs. Results: A total of 2058 college students participated in this study. Students who were willing to get COVID-19 vaccines had significantly higher BAS-2 scores than did those who were unwilling to receive a vaccine (3.61 ± 0.84 vs. 3.34 ± 0.92, *p* < 0.001). A hierarchical multiple logistic regression analysis was performed to test the association between body appreciation and COVID-19 vaccine intentions when controlling for other covariates; elevated BAS-2 scores were associated with greater willingness to receive COVID-19 vaccines (OR = 1.250, 95%CI: 1.112–1.406, *p* < 0.001), independent of other significant influences. Conclusion: Our study was the first to reveal that body appreciation is a significant factor related to college students’ COVID-19 vaccination intentions. Public health interventions designed to improve people’s body-appreciation levels may help in efforts to promote universal immunization.

## 1. Introduction

Following the initial manufacture of coronavirus disease 2019 (COVID-19) vaccines, numerous studies have arisen to investigate factors associated with people’s willingness to receive a COVID-19 vaccine [1,2,3]. Recent studies have found that COVID-19 vaccine uptake intentions are associated with certain demographic characteristics (e.g., living in an urban area, older age, female gender, higher income and educational levels, status as a healthcare worker, and higher perceived personal risk of being infected) as well as greater knowledge of and more favorable attitudes toward COVID-19 vaccine safety and efficacy [4,5,6,7,8,9,10,11,12].

Given that vaccinations are a health preventive measure designed to protect the physical body [13], willingness to be vaccinated may also be related to people’s thoughts about, attitudes to, and perceptions of their body [14]. Previous studies have found that positive body-image attitudes reflecting body appreciation (i.e., acceptance, respect, protection, and love towards one’s body), were associated with individuals’ health-protective behaviors, including screening for cancer, sun protection, limiting amount of alcohol use, engagement in healthy eating practices, exercise frequency, the pursuit of medical attention when required, engagement in meditation, and use of appropriate oral health and sexual health behaviors [15,16,17]. In addition, previous studies have found influenza vaccinations are associated with perceived stress and self-esteem [18], both of which are strongly associated with body appreciation [17,19,20]. Similarly, vaccine acceptance has been linked to lower levels of depression and anxious symptoms [21,22], each of which is closely related to greater body appreciation [23,24]. Together, such findings suggest that body appreciation is a plausible factor associated with people’s COVID-19 vaccination intentions that can operate either directly or indirectly through its relationships with levels of self-esteem, perceived stress, and/or emotional distress.

To date, most of the relevant research has focused on beliefs, attitudes or perceptions related to COVID-19 vaccines, but no studies, to date, have examined the relationship between people’s attitudes towards their body (i.e., body image) and COVID-19 vaccination intentions. In addition, compared with older subpopulations, young adults (e.g., college students) are more likely to experience elevations in appearance anxiety and body image disturbances, which can have negative repercussions for their mental and physical well-being [25,26,27] in tandem with a reduced likelihood of being vaccinated. Given that associations between body image attitudes and health-related behaviors reflecting COVID-19 vaccination intentions have not been examined within this population at risk of remaining unvaccinated, we examined the association between body appreciation and future COVID-19 vaccine uptake intentions among college students.

## 2. Materials and Methods

### 2.1. Setting and Sample

This cross-sectional study was conducted among Chinese college students between December 2020 and January 2021, using a snowball sampling method. Snowball sampling is a non-probabilistic sampling approach in which existing subjects provide referrals to recruit others from their own social networks to participate in the same study [28]. Because of its cost-effectiveness and efficiency in identifying targeted participants, snowball recruiting has been used widely, especially during the COVID-19 pandemic, during which it has not been feasible to collect data based on face-to-face interviews [29,30,31,32].

Following previous studies [33,34,35], we designed an online questionnaire using the Questionnaire Star application. We invited influential academic staff, including University presidents, faculty deans and department heads, to forward the online questionnaire to their students and encourage them to participate in this study via WeChat, a widely used social communication application with more than 1 billion users in China [4]. To be eligible, participants were required: (1) to be undergraduate students; (2) to be ethnic Chinese; and (3) to have adequate comprehension of the research purpose and contents of the assessment. Exclusion criteria included presence of major physical diseases affecting cardiovascular, respiratory, digestive, hematological, endocrine, urinary, connective tissue, and/or nervous systems [36] or psychiatric disorders that had been formally diagnosed by psychiatrists. This study was approved by the Institutional Review Board (IRB) of Beijing Anding Hospital. Online written informed consent was obtained. Consent was also required from legal guardians if participants were under 18 years of age.

### 2.2. Assessment Tools

We collected basic sociodemographic and health-related information related to age, gender (female/male), undergraduate year (first/second/third/fourth/fifth year), academic major (health-related/other), residence (urban/rural), perceived health status (poor/fair/good), and body mass index (BMI). Additional questions were included to solicit further information about participants’ and family members’ status regarding previous COVID-19 infection, current worry-level about being infected with COVID-19, and awareness of COVID-19 vaccines. Additionally, attitudes toward COVID-19 vaccines were assessed in relation to participants’ beliefs about the safety and efficacy of COVID-19 vaccines. In previous studies [4,5,37], participants’ intentions to have a COVID-19 vaccine were examined with a single question: “Are you willing to get COVID-19 vaccines in the future?” which included a dichotomous response option (yes/no). 

Body appreciation was assessed with the 10-item Body Appreciation Scale-2 (BAS-2), Chinese version [38,39]. The BAS-2 assesses acceptance of, favorable opinions toward, and respect for one’s body. Items are rated on a 5-point Likert scale (i.e., 1 = never, 2 = seldom, 3 = sometimes, 4 = often, 5 = always), summed and averaged to obtain an overall body appreciation score. Higher scores reflect greater body appreciation. The Chinese version of the BAS-2 fully replicates the original factor structure of the scale, and has satisfactory psychometric properties, such as Cronbach’s alpha coefficients of α = 0.89 and α = 0.86 in female and male Chinese college students, respectively [38].

### 2.3. Data Analysis

Data were analyzed using the IBM Statistical Package for Social Sciences (SPSS) program, version 23.0. Participants were split into two groups according to their willingness to have COVID-19 vaccines in the future. A P-P plot was used to test the normality of continuous variables. To compare differences in demographics, clinical characteristics, and body appreciation between willing versus unwilling groups, Chi-square tests, Fisher’s exact tests, two-sample independent t tests and Mann-Whitney U Tests were used, as appropriate. To test the independent association between BAS-2 score and willingness to receive COVID-19 vaccines in the future, a hierarchical logistic multiple regression analysis with two blocks was conducted. Willingness versus unwillingness to receive future COVID-19 vaccines was the dependent variable. Measures, aside from BAS-2 scores, on which willing versus unwilling groups significantly differed in univariate analyses were entered first as independent variables in Block 1 of the model. Subsequently, BAS-2 scores were entered in Block 2 of the model to evaluate the unique relationship of body appreciation with the dependent measure, after the potentially confounding effects of variables having significant associations with willingness to receive future COVID-19 vaccines (i.e., covariates) had been controlled for. The level of significance for analyses was set at *p* < 0.05 (two-tailed).

## 3. Results

### 3.1. Demographic Characteristics of the Sample 

A total of 2282 college students were invited to participate in this study. Of these, 2058 college students (1393 women and 665 men) from 25 of mainland China’s 32 provinces met the study entry criteria and were included in data analyses. Table 1 provides a summary of demographic characteristics of the overall sample as well as willing versus unwilling COVID-19 vaccination subgroups. 

### 3.2. Correlates of Intentions toward Future COVID-19 Vaccine Uptake

**Figure 1:**
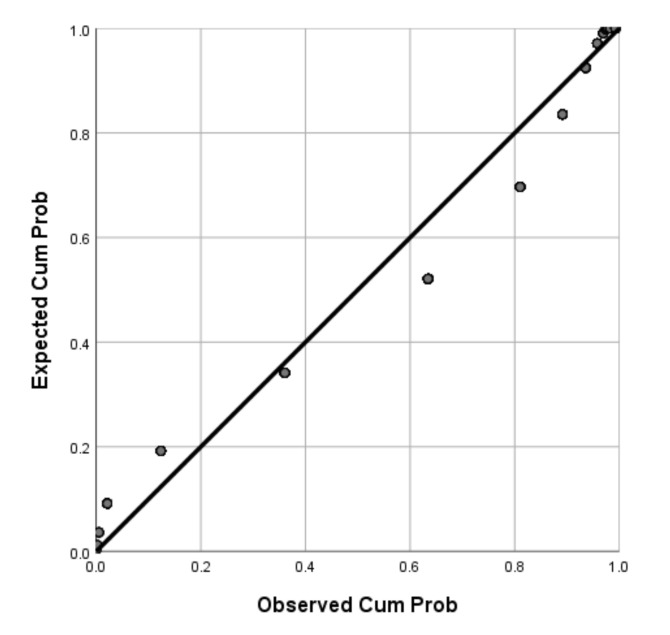
P-P plot of Age.

**Figure 2:**
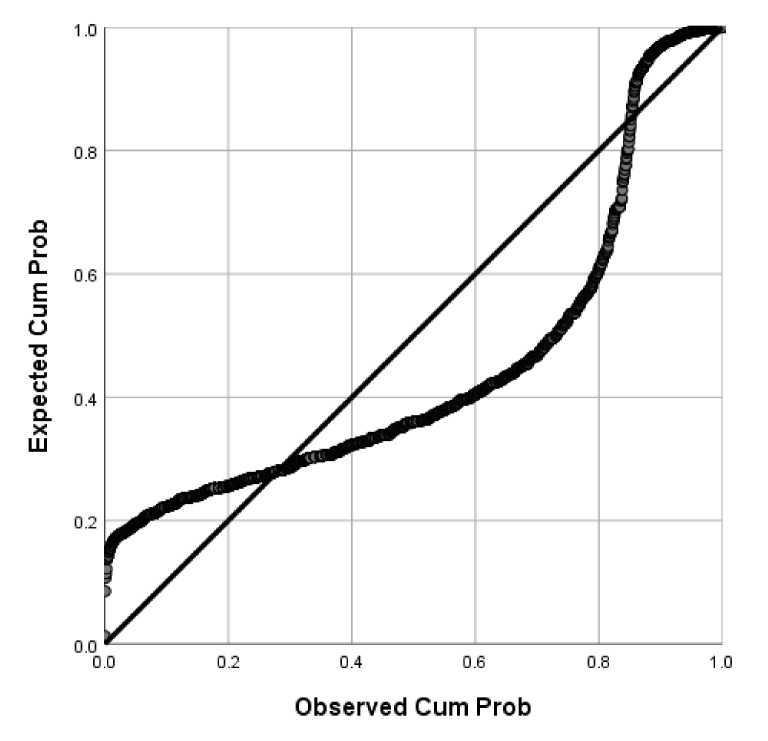
P-P plot of BMI.

**Figure 3:**
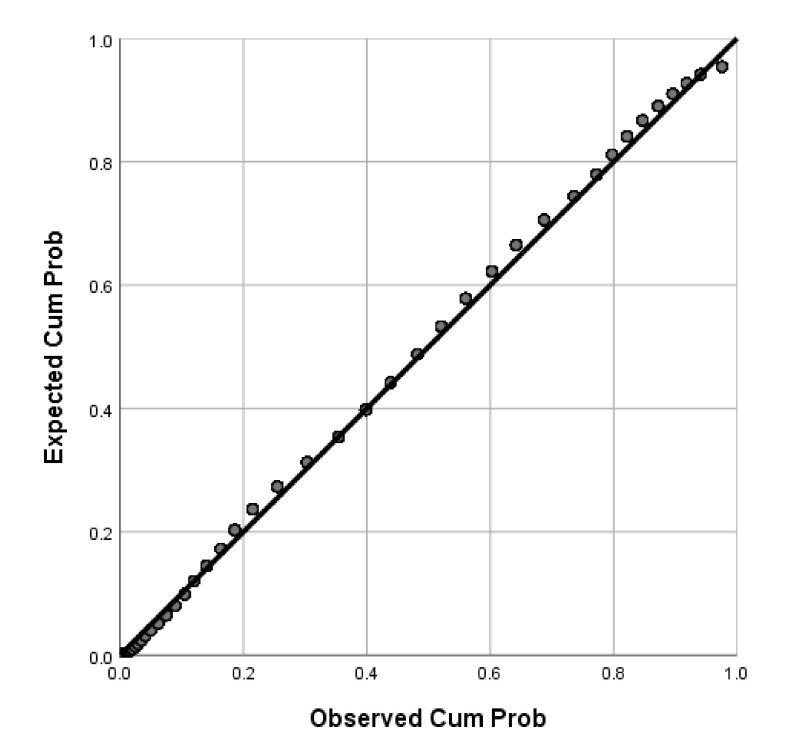
P-P plot of BAS-2 score.

Of the participants, 71.04% (95%CI: 69–73%) were willing to receive COVID-19 vaccines in the future. P-P plots showed that the distributions of age and BMI were skewed while BAS-2 total scores were normally distributed (Figure 1, Figure 2 and Figure 3). Thus, Mann-Whitney U Tests were used to compare age and BMI and a two-sample independent t test was used to compare the BAS-2 total score difference between the two groups. As shown in Table 1, univariate analyses revealed significant differences between students who were willing to receive COVID-19 vaccines in the future versus those unwilling to be vaccinated, in terms of study major, place of residence, COVID-19 vaccine-related knowledge and attitudes, and BAS-2 scores. Specifically, participants who were willing to receive COVID-19 vaccines in the future were more likely to be pursuing a major degree in a health-related discipline (*p* < 0.001), to live in an urban area (*p* = 0.013), to have heard about COVID-19 vaccines previously (*p* < 0.001), to believe COVID-19 vaccines could provide protection (*p* < 0.001), to believe COVID-19 vaccines were safe (*p* < 0.001), and to report a higher BAS-2 score (*p* < 0.001).

### 3.3. Test of the Relationship between Body Appreciation and COVID-19 Vaccine Intentions 

To test the independent association between body appreciation and COVID-19 vaccine intentions, we performed hierarchical logistic multiple regression analysis. All measures, aside from BAS-2 scores, on which willing versus unwilling groups showed significant differences in univariate analyses, were entered first as covariates in Block 1 of the model (i.e., health-related major, residence, having heard about COVID-19 vaccines previously, belief that COVID-19 vaccines provide protection, belief that COVID-19 vaccines are safe). As shown in Table 2, after controlling for the effects of these factors in the model, BAS-2 score (entered in Block 2) was a significant factor associated with students’ COVID-19 vaccine intentions (OR = 1.250, 95%CI: 1.112–1.406, *p* < 0.001): higher body-appreciation scores were significantly associated with increased willingness to take up COVID-19 vaccines in the future.

## 4. Discussion

This was the first study to document a relationship between college students’ body-appreciation levels and their willingness to receive COVID-19 vaccines in the future. Specifically, we found that Chinese college students who were willing to receive COVID-19 vaccines in the future had significantly higher overall body-appreciation scores than did those who were unwilling to receive vaccines (3.61 vs. 3.34, OR = 1.250, 95%CI: 1.112–1.406, *p* < 0.001), even after controlling for several factors that have been widely shown to be associated with COVID-19 vaccine uptake, including COVID-19 vaccine-related knowledge and attitudes, place of residence, and study major [4,5,6,7,8,9,10,11,12].

The relationship between body appreciation and vaccine uptake intentions has not been studied previously. Some studies have found that people with higher body-appreciation levels are more likely to engage in health-protective behaviors, such as cancer screening, purchasing sun protection products, seeking early medical attention, and exercising more frequently [15,16]. Such persons are more attentive to their body’s needs and feel more connected to their body; consequently, high levels of body appreciation may help to foster and maintain good physical health [40]. In contrast, reduced respect for and appreciation of one’s body may be a barrier to engaging in appropriate medical care [41]. Since vaccination is a typical health-protective behavior that helps to protect against infectious diseases [13], it is reasonable to assume that people with higher body-appreciation levels are more willing to seek COVID-19 vaccines in the future compared to their peers with lower body-appreciation levels, a contention that was confirmed by our findings. Among people who love and respect their bodies, an array of actions designed to protect and improve health are evident [15,16,17]. Another possible reason that body-appreciation elevations corresponded with increased willingness to be vaccinated is that people who have favorable attitudes toward their body also tend to have higher levels of self-esteem, in addition to lower levels of perceived stress and emotional distress [21,22]. Together, these data suggest that higher body-appreciation levels may be a marker for comparatively better overall mental health, which is also positively associated with COVID-19 vaccination intentions.

Aside from body appreciation, the logistic regression analysis indicated willingness to be vaccinated was related to having heard about COVID-19 vaccines previously as well as the belief that COVID-19 vaccines are safe. These findings add to a growing literature that underscores the importance of vaccine-availability awareness, vaccine safety, and perceived vaccine benefits as important factors associated with willingness to pursue COVID-19 vaccinations [4,5,6,7,8,9,10,11,12].

Results of this study have potentially important implications for both research and clinical practice. Specifically, our findings provided evidence for the identification of a previously undocumented factor, body appreciation, as a significant correlate of COVID-19 vaccine-uptake intentions within a group (i.e., young adults) that is at risk for remaining unvaccinated. In addition to considering people’s attitudes toward COVID-19 vaccines, our results underscored the potential importance of attending to people’s attitudes toward their own body as a key factor associated with their decisions to seek or avoid COVID-19 vaccines. As such, our findings illustrated how positive body image may play a possibly crucial role in the embrace of specific protective health behaviors related to immunization. The robust association between body appreciation and vaccination intentions in our sample also provides a foundation for the development of interventions and initiatives that may have utility in increasing compliance with recommended vaccination protocols [16]. 

From a clinical practice perspective, during the COVID-19 pandemic it is critical to prevent further spreading of COVID-19. Vaccines provide a timely solution that can control and ultimately end the pandemic. Thus, increasing people’s willingness to receive COVID-19 vaccines is indispensable for achieving universal immunization. According to recent surveys, around 25% of people worldwide are unwilling to receive COVID-19 vaccines [4,8,12,42]. As of the middle of 2021, more than half of the general population worldwide remains unvaccinated [43]. Apart from misinformation, the shortage of vaccines in many countries and the distrust of COVID-19 vaccine safety and efficacy, our main findings suggest that low levels of body appreciation may be a barrier to the future uptake of COVID-19 vaccines. Therefore, our findings provide an innovative perspective on strategies that have potential utility in motivating the unvaccinated to pursue vaccinations. Apart from providing consistent science-based education about the safety and efficacy of COVID-19 vaccines [4,8], interventions designed to increase body-awareness, self-compassion, cognitive dissonance in the pursuit of unhealthy body-related behaviors, and social media publicity directed at fostering a more positive body image may help to promote people’s appreciation of their bodies [44] and encourage COVID-19 vaccination uptake among those who are currently unwilling to take this step in protecting their health.

Despite its novel findings and potential implications, this study has several limitations that should be acknowledged. First, because it focused on one at-risk group (i.e., young adults) and a non-random sample of Chinese college students was assessed toward this end, findings cannot be generalized to all college students in China, or other age groups, such as children, middle aged adults, and older adult groups, less educated groups, or to samples from other cultures. Second, although response burdens on unpaid research volunteers were not excessive, some potential correlates of COVID-19 vaccine uptake intentions (e.g., family income, parents’ education, personality, psychopathology) were not assessed. Therefore, it is possible that unmeasured factors also affected vaccination intentions. Third, because only a very small number of students and/or their family members had been infected with COVID-19, the impact of exposure to COVID-19 on vaccination intentions could not be determined. Finally, this was a cross-sectional, anonymous study; therefore, we were unable to follow up on participants to assess prevalence of vaccination uptake and assess the causal relationship between body appreciation and COVID-19 vaccination intentions.

## 5. Conclusions

In conclusion, our study found that, apart from attitudes toward COVID-19 vaccine safety and efficacy, Chinese college students with higher body-appreciation levels were more willing to receive COVID-19 vaccines in the future. This finding provides an important impetus for extension to other populations, and for public health strategies to promote and increase people’s love and respect for their own bodies as a means to bolster willingness to be vaccinated.

## Figures and Tables

**Table 1:** Differences between students who are willing versus unwilling to receive a COVID-19 vaccine in the future.

Categorical Variables	Total		Unwilling to Get COVID-19 Vaccine		Willing to Get COVID-19 Vaccine		Univariate Analyses		
	(*N* = 2058)		(*N* = 596)		(*N* = 1462)				
	*N*	%	*N*	%	*N*	%	χ^2^	df	*p*
Demographic information									
Male gender	665	32.31	192	32.21	473	32.35	0.004	1	0.952
Grade									
First	1337	64.97	337	56.54	960	65.66	9.491	4	0.05
Second	393	19.10	134	22.48	259	17.72			
Third	172	8.36	47	7.89	125	8.55			
Fourth	79	3.84	15	2.52	64	4.38			
Fifth	77	3.74	23	3.86	54	3.69			
Health-related major	1158	56.27	258	43.29	900	61.56	57.439	1	**<0.001**
Residence (Urban)	829	40.28	215	36.07	614	41.99	6.176	1	**0.013**
Perceived health status as good	1580	76.77	467	78.36	1113	76.13	1.178	1	0.278
COVID-19 related information									
Had been infected with COVID-19 or had infected family members	11	0.53	3	0.50	8	0.55	-^a^	-^a^	0.999
Worried about being infected with COVID-19									
No	329	15.99	109	18.29	220	15.05	4.653	2	0.098
Fair	1327	64.48	383	64.26	944	64.57			
Very much	402	19.53	104	17.45	298	20.38			
Knowledge, attitude, belief towards COVID-19 vaccine									
Heard about COVID-19 vaccines previously	1745	84.79	425	71.31	1320	90.29	118.257	1	**<0.001**
Believe COVID-19 vaccines could provide protection	978	47.52	191	38.51	787	53.83	80.561	1	**<0.001**
Believe COVID-19 vaccines are safe	443	21.53	61	10.23	382	26.13	63.316	1	**<0.001**

Continues Variable	Mean	SD	Mean	SD	Mean	SD	t/Z	df	*p*
Age (years)	19.89	2.17	19.87	1.99	19.89	2.23	−1.702	-^b^	0.089
BMI	23.52	7.74	23.47	7.97	23.54	7.65	−1.672	-^b^	0.095
BAS-2	3.53	0.87	3.34	0.92	3.61	0.84	−6.379	2056	**<0.001**

Bolded values: *p* < 0.05; COVID-19: Coronavirus Disease 2019; BAS-2: Body Appreciation Scale-2; SD: standard deviation; BMI: Body Mass Index; ^a^ Fisher’s Exact Test; ^b^ Mann-Whitney U Test.

**Table 2:** Hierarchical logistic multiple regression model of factors that were associated with willingness to receive a COVID-19 vaccine in the future.

	Hierarchical Logistic Multiple Regression Analysis		
	*p* Value	OR	95% CI
Block 1			
Health-related major	<0.001	0.584	0.475–0.718
Residence (Urban)	0.012	1.306	1.059–1.610
Heard about COVID-19 vaccines previously	<0.001	2.576	1.981–3.349
Believe COVID-19 vaccines can provide protection	<0.001	1.758	1.418–2.180
Believe COVID-19 vaccines are safe	<0.001	2.200	1.623–2.982
Block 2			
Health-related major	<0.001	1.636	1.328–2.016
Residence (Urban)	0.011	1.314	1.065–1.621
Heard about COVID-19 vaccines previously	<0.001	2.468	1.894–3.214
Believe COVID-19 vaccines could provide protection	<0.001	1.727	1.392–2.143
Believe COVID-19 vaccines are safe	<0.001	2.149	1.584–2.916
BAS-2 mean score	<0.001	1.250	1.112–1.406

Bolded values: *p* < 0.05; CI: confidential interval; OR: odds ratio; BAS-2: Body appreciation scale-2.

## Data Availability

The data presented in this study are available on request from the corresponding author. The data are not publicly available due to participants’ privacy.
